# Clustering of 24‐H Movement Behaviours and Its Associations With Eating Behaviours and Adiposity Among Mongolian Preschool Children: A Cross‐Sectional Study

**DOI:** 10.1111/cch.70084

**Published:** 2025-04-16

**Authors:** Ankhmaa Byambaa, Rachel A. Jones, Kar Hau Chong, Oyundelger Dechinjamts, Bayasgalan Jambaldorj, Anthony D. Okely

**Affiliations:** ^1^ School of Social Sciences, Faculty of the Arts, Social Sciences and Humanities University of Wollongong Wollongong New South Wales Australia; ^2^ National Centre for Public Health Ulaanbaatar Mongolia

**Keywords:** behaviour, eating, obesity, physical activity, sleep

## Abstract

**Background:**

Obesity‐related behaviours such as physical activity, sedentary behaviour, sleep, screen time and diet often cluster in children. There is limited evidence on the clustering of movement behaviours among young children from low‐ and middle‐income countries. This paper reports how 24‐h movement behaviours cluster in Mongolian preschool children and their associations with eating behaviours and adiposity.

**Methods:**

Cross‐sectional study involved 201 children aged 3–4 years attending kindergartens in urban and rural areas of Ulaanbaatar city, Tuv and Uvurkhangai provinces, Mongolia. Children wore accelerometers to measure physical activity, sedentary behaviour and sleep. Parents completed a questionnaire to report screen time and eating behaviours. To derive clusters, hierarchical and k‐means cluster analyses were performed sequentially. Associations between clusters, eating behaviours and BMI z‐score were analysed using ANOVA. Logistic regression was applied to estimate the odds of being overweight depending on cluster membership.

**Results:**

Three clusters were identified: *All‐rounders*, *Non‐active Sleepers* and *Screeners*. We found that around half the children exhibited at least one unhealthy behaviour and were classified into clusters with a mix of healthy and unhealthy behaviours. The clusters did not differ by sociodemographic characteristics. No significant association was found between cluster membership and BMI z‐score of children. The cluster at highest risk for being overweight was *Screeners* (odds ratio = 1.7, 95% CI: 0.67–4.33), who exhibited two obesogenic behaviours simultaneously, screen time for > 4 h per day and regular consumption of unhealthy snacks (53%, *p* = 0.033) and sugary drinks (53%, *p* = 0.014).

**Conclusion:**

Obesity prevention measures should begin in early childhood and target high‐risk clusters, considering the co‐occurrence of healthy and unhealthy behaviours. More research is needed in Mongolia to provide evidence for obesity prevention policies and inform targeted interventions to promote healthy behaviours from a young age.

**Summary**

- By investigating the lifestyle behaviours of young children in Mongolia, where the prevalence of overweight and obesity is high, this study provides crucial insights into an under‐researched topic.
- Our findings show that Mongolian preschoolers can be grouped into three distinct clusters based on their 24‐h movement behaviours. The clusters were not exclusively healthy or unhealthy; instead, they contained a mix of behaviours.
- ‘Sedentary‐snacking’ behaviour is prevalent among Mongolian preschoolers, and a high‐risk factor for overweight and obesity.
- Further research is necessary to confirm and expand upon these results by including a diverse range of sociodemographic characteristics and health indicators while employing longitudinal design to ensure a more comprehensive understanding of children's lifestyle behaviours in Mongolia.

## 1 Introduction

Childhood obesity is a pressing global health issue, with incidence rates quadrupling over the past three decades (Phelps et al. 2024; World Health Organization 2024). Studies indicate that 11.3%–12.8% of children under five in Mongolia are overweight. This is considered a ‘high’ prevalence rate compared to other Asian countries, calling for public health prioritization (National Center for Public Health of Mongolia 2024; National Statistics Office of Mongolia, UNICEF 2023; Norov et al. 2023). Maintaining optimal levels of physical activity (PA), and sleep duration, while limiting sedentary screen time (collectively known as movement behaviours) has been recognized as a key strategy to prevent childhood obesity (World Health Organization 2016). Systematic reviews have reported favourable associations between meeting 24‐h movement behaviour guidelines and health outcomes in children (Rollo, Antsygina, and Tremblay 2020; World Health Organization 2019). Conversely, inadequate levels of movement behaviours from an early age may be detrimental to children's development and can place them at higher risk of becoming overweight or obese (World Health Organization 2019).

Cluster analysis is a useful method for exploring lifestyle patterns as it groups participants into mutually exclusive groups based on similar characteristics (Hair et al. 2010). When applied to health‐related behaviours, it helps identify high‐risk groups and facilitates a nuanced exploration of how behaviours co‐occur within specific groups (D'Souza et al. 2020; Leech, McNaughton, and Timperio 2014; Gubbels, van Assema, and Kremers 2013). Systematic reviews indicate that clusters characterized by a mix of healthy and unhealthy behaviours, such as high PA and high sedentary behaviour (SB), were more prevalent than clusters exhibiting exclusively healthy (e.g., high PA/low SB) or unhealthy behaviours (e.g., low PA/high SB) (D'Souza et al. 2020; Leech, McNaughton, and Timperio 2014). However, little is known about the clustering of movement behaviours among children under the age of five, particularly in low‐ and middle‐income countries (LMICs) (D'Souza et al. 2020; Leech, McNaughton, and Timperio 2014; Gubbels, van Assema, and Kremers 2013; Mello et al. 2023).

A risk factor for childhood obesity is excessive consumption of discretionary foods, which are high in calories but low in nutritional value (Davison and Birch 2001). However, previous studies have focused on dietary intake (i.e., what is eaten) rather than eating behaviours, such as the contextual aspects of food consumption (i.e., where, how and with whom food is consumed) (Gubbels, van Assema, and Kremers 2013). Existing research shows that watching television during mealtimes increases the odds of children drinking sugar‐sweetened beverages and consuming less healthy and more processed foods (Avery, Anderson, and McCullough 2017). Understanding eating behaviours of children can provide valuable insights into understanding childhood obesity.

Therefore, the aim of this study was twofold: to identify clusters of movement behaviours among preschool children in Mongolia and to examine their associations with eating behaviours and adiposity. This will support the development of targeted strategies and interventions for promoting the health of young children, especially the prevention of obesity in other LMICs.

## 2 Methods

### 2.1 Study Design and Population

This was a cross‐sectional study of preschool‐aged children from the Mongolian site of the SUNRISE International Study of Movement Behaviours in the Early Years (https://sunrise‐study.com) (Okely et al. 2021).

The SUNRISE Mongolia study consists of two phases: pilot and main phase. The pilot phase was conducted from December 2021 to April 2022 and involved 101 participants from five different early childhood education settings (referred to as kindergartens) of Ulaanbaatar city and Tuv province. The main phase of the study is ongoing, with data collected from 100 participants across four kindergartens between March and May 2023. In total, data collection involved nine kindergartens in urban and rural areas of Ulaanbaatar city (*n* = 5), Tuv province (*n* = 2) and Uvurkhangai province (*n* = 2).

Participant recruitment employed a convenience cluster sampling approach, where kindergartens served as clusters. Directors of selected kindergartens were invited to participate, followed by meetings with parents or caregivers (referred to as parents) to explain the study details. Parents interested in enrolling their child were provided with an information sheet and consent form. Children with parental informed written consent were recruited, with a maximum of 25 children from each kindergarten. In cases where the number of eligible children with written consent exceeded the required number of participants, random selection was employed. Eligibility criteria included being of appropriate age (3–4‐years old) and able to wear an accelerometer.

### 2.2 Measurements

#### 2.2.1 Adiposity

Children's height (cm) and weight (kg) were measured with calibrated scales (SECA 876, Germany) and a height measuring board (UNICEF) following the World Health Organization (WHO) protocols. BMI‐for‐age Z‐score (BMI z‐score) was calculated using WHO AnthroPlus v1.0.4 software (WHO, Geneva, Switzerland). Child's body weight status was classified based on WHO growth standards (World Health Organization n.d.‐a; World Health Organization n.d.‐b).

#### 2.2.2 24‐H Movement Behaviours

PA, sedentary time and sleep were assessed using waist‐worn ActiGraph GT3X+ and wGT3X‐BT devices (Actigraph LLC). Accelerometers were initialised to record data at 30 Hz. Children wore the monitors for five consecutive days, excluding water‐based activities. Raw data were downloaded from the devices using ActiLife software (version 6.12.1) and processed in R (version 4.2.1) using an automated script (Cliff et al. 2024). Briefly, raw data files were converted into counts per second format using the ‘activityCounts’ package, which were then collapsed into 60‐ and 15‐s epochs for sleep and PA analysis, respectively, using the ‘PhysicalActivity’ package. Sleep periods were identified using the decision‐tree‐based algorithm in the ‘PsycActBedRest’ package validated for preschool children (Tracy et al. 2021). Nonwear during sleep was defined as ≥ 90 min of consecutive zero counts with up to 2 min of nonzero interruption (Choi et al. 2011). Any sleep periods identified between 10 AM and 7 PM were categorised as daytime naps and included in the calculation of total sleep duration. For waking hours data, any period(s) of ≥ 20 min of consecutive zero counts were marked as nonwear, with the remaining awake time data categorised as SB (< 200 counts/15 s), light‐ to vigorous‐intensity PA (≥ 200 counts/15 s) (Pate et al. 2006; Pate et al. 2015). A valid day of accelerometer data was defined as ≥ 10 h of wear time during the day and ≥ 160 min of total sleep periods (Migueles et al. 2017; Roman‐Viñas et al. 2016).

Screen time was proxy‐reported by parents via the following question: (Okely et al. 2021) *On a typical 24‐hour period in the past week, how much time did the child who is participating in this study spend using any electronic screen device such as smart phone, tablet, video game, or watch TV or movies, videos on the internet while they were sitting or lying down?*

#### 2.2.3 Eating Behaviours

Eating behaviours were evaluated using five questions answered by parents (Table S1) (Okely et al. 2021). These included two on family meal dynamics (e.g., ‘How often do you sit with your child during meals?’), one on the child's frequency of TV or electronic screen device usage during meal or snack time, and two on the child's frequency of consuming unhealthy snacks (e.g., chips, candies and pastries) between meals, and sugary drinks (e.g., soda and soft drink).

Parents responded to each question using a 5‐point Likert scale, i.e., ‘never’, ‘rarely’, ‘once a week’, ‘most days’ and ‘everyday’, with an additional ‘Do not know’ option treated as a missing data. Response to each question was assigned scores from 0 to 4 points with higher score indicating more favourable behaviour. For example, response ‘everyday’ for questions on family meal dynamics received 4 points, and ‘never’ received 0 points. On the other hand, response ‘never’ for the last three questions on the frequency of screen device usage and snacking received 4 points, and ‘everyday’ received 0 points. The total scores of the five questions were calculated (ranging between 0 and 20 points), with a higher score indicating more favourable eating behaviour.

#### 2.2.4 Demographic Characteristics

In addition to screen time and eating behaviours, parents were also asked to report the child's sociodemographic characteristics (date of birth and sex), and the highest level of parental education completed by a member of household. Participants were categorised by sector and location based on the geographical placement of the kindergartens they attended. Sector refers to Mongolia's official administrative divisions, distinguishing between urban areas (capital city of Ulaanbaatar and provincial central towns) and rural areas (all other towns). Location is a study‐specific categorization, dividing participants into those from Ulaanbaatar and those from provincial towns.

All questionnaires were translated into Mongolian and distributed to parents to complete at home. Although the questionnaires have not been validated, they have been shown to be practical and culturally appropriate for use in Mongolia and countries of varying income levels (Byambaa et al. 2024; Delisle Nyström et al. 2020; Hossain et al. 2021).

### 2.3 Statistical Analysis

All statistical tests were performed using IBM SPSS (version 28.0.1.0) and Jamovi software (version 2.2.5). Participants needed a minimum of 1 day of valid accelerometer data and complete dataset for other variables to be included in the analyses.

Prior to cluster analysis, z‐scores were computed by standardizing each movement behaviour variable (PA, SB, screen time and sleep). Participants with z‐score > 3 standard deviations were excluded from the analysis. Hierarchical and nonhierarchical cluster analysis were employed to identify clusters with similar movement behaviour patterns (Hair et al. 2010; D'Souza et al. 2021). The hierarchical analysis with Ward's method and the squared Euclidean distance measure determined an optimal three‐cluster solution, as evidenced by the dendrogram plot (Figure S1). This optimal cluster configuration informed the subsequent nonhierarchical k‐means cluster analysis. The k‐means analysis employing MacQueen algorithm and a maximum number of 99 iterations yielded stability in the three‐cluster solution. The final cluster solution was made based on convergence iterations, interpretability of the results and the proportion of the study population in each cluster (Figure S2) (Hair et al. 2010).

Descriptive characteristics of participants were calculated and summarized by clusters. Differences in sociodemographic characteristics (child's sex, sector, location and parental education level) between clusters were tested using Chi‐squared test. Distribution of unfavourable eating behaviours across clusters was tested by Chi‐squared test. Associations of cluster membership with eating behaviours score and BMI z‐score were analysed using ANOVA test, while the associations with weight status (overweight vs. nonoverweight) were assessed using logistic regression analysis, both unadjusted and adjusted for sex and parental education level. Statistical significance was set at *p* < 0.05.

## 3 Results

A total of 201 children (mean age = 4.5 years) participated in the study. After excluding outliers (≥ 3 SD) (*n* = 7) and participants with incomplete data (*n* = 18), 176 children were included in the final analysis (Table 1). The number of boys and girls were comparable, with slightly more children from urban settings. On average, children spent almost 3 h engaging in PA, 11.5 h sleeping, 8 h being sedentary and over 2 h of screen time.

**TABLE 1:** Descriptive characteristics of the participants.

	Total, *n*	Frequency (%)/mean (SD)
Sex		
Boys	176	88 (50%)
Girls		88 (50%)
Sector		
Urban	176	96 (54.5%)
Rural		80 (45.5%)
Location		
Ulaanbaatar	176	107 (60.8%)
Province		69 (39.2%)
Highest level of parental education completed by a member of household		
Full secondary and below	173	45 (26%)
Tertiary and higher		128 (74%)
Movement behaviours		
Physical activity (min/day)	176	168 (40)
Sleep (min/day)	176	713 (92)
Screen time (min/day)	176	131 (85)
Sedentary behaviour (min/day)	176	486 (60)
Eating behaviours		
Parents do not usually sit with child during meals	168	16 (9.5%)
Family do not usually have main meal together	167	18 (10.8%)
Regular* use of TV or other screen during meals	160	80 (50%)
Regular* consumption of unhealthy snacks	163	59 (36.2%)
Regular* consumption of sugary drink	167	51 (30.5%)
Eating behaviours total score**	170	13 (4)
Weight status	176	
Severely wasted		1 (0.6%)
Normal		134 (76.1%)
Possible risk of overweight		29 (16.5%)
Overweight or obese		12 (6.8%)
BMI z‐score		0.39 (0.99)

* Regular use or consumption refers to using TV or consuming snacks and sugary drinks every day and most of days.

** Eating behaviours total score range is 0–20 points.

### 3.1 Clustering of Movement Behaviours

Three distinct clusters of movement behaviours were identified (Table 2). Cluster 1 (*n* = 85, 48.3%), labelled as *All‐rounders*, displayed the highest levels of PA coupled with minimal screen time and sleep duration. Cluster 2 (*n* = 51, 29%), labelled as *Non‐active Sleepers*, displayed the lowest levels of PA and SB, along with the highest sleep duration. Cluster 3 (*n* = 40, 22.7%), labelled as *Screeners*, had the opposite dynamic with moderate levels of PA and sleep, yet had an exceptionally high screen time. The final cluster centres by z‐scores are shown in Figure S3. There was no significant difference between clusters by sociodemographic variables (Table S2).

**TABLE 2:** Mean values (standard deviation) of time spent in movement behaviours by clusters.

Cluster	Number of participants	PA (min/day)	Sleep (min/day)	Screen (min/day)	SB (min/day)
C1 All rounders	85	182 (34)	673 (64.5)	85 (46)	508 (46)
C2 Non‐active sleepers	51	136 (33)	808 (79.5)	112 (52)	424 (47)
C3 Screeners	40	177 (40)	678 (66.9)	252 (63)	517 (45)

Abbreviations: PA, physical activity; SB, sedentary behaviour.

### 3.2 Association of Cluster Membership With Eating Behaviours

The eating behaviours score for *Screeners* was significantly lower (*p* < 0.001) than that for *All‐rounders* and *Non‐active Sleepers* (Table 3). *Screeners* comprised a higher proportion of children who regularly consumed sugary drinks (*p* = 0.014) and unhealthy snacks (*p* = 0.033) compared to the other two clusters (Table 4).

**TABLE 3:** Differences in children's adiposity and eating behaviour scores across clusters, mean values (standard deviation).

	C1 *All‐rounders*	C2 *Non‐active Sleepers*	C3 *Screeners*	*p*‐value
BMI z‐score	0.43 (0.9)	0.42 (0.9)	0.28 (1.3)	0.799
Eating behaviours total score	13.4 (4)	13.8 (3)	11.1 (3)	< 0.001

Abbreviation: BMI, body mass index.

**TABLE 4:** Unfavourable eating behaviours distribution across clusters.

Unfavourable eating behaviours	C1 *All‐rounders*	C2 *Non‐active Sleepers*	C3 *Screeners*	*p*‐value
Parents do not usually sit with child during meals	7%	6%	18%	0.154
Family do not usually have main meal together	7%	6%	23%	0.085
Regular* use of TV or other screen during meal	43%	40%	66%	0.156
Regular* consumption of unhealthy snacks	42%	30%	53%	**0.033**
Regular* consumption of sugary drink	24%	32%	53%	**0.014**

*Note:* Significant relationships are bolded (*p* < 0.05).

* Regular use or consumption refers to using TV or consuming snacks and sugary drinks every day and most of days.

### 3.3 Association of Cluster Membership With Adiposity

No significant difference was found in children's BMI z‐scores across clusters (Table 3). Although not statistically significant, *All‐rounders* appeared less likely to be overweight compared with *Non‐active Sleepers* (adjusted odds ratio [adjOR] = 1.57, 95% CI: 0.64–3.83) and *Screeners* (adjOR = 1.7, 95% CI: 0.67–4.33) (Table 5).

**TABLE 5:** Results of logistic regression analysis for overweight prediction.

	Unadjusted model		Adjusted model	
	OR (95% CI)	*p*‐value	OR (95% CI)	*p*‐value
C1 *All‐rounders*	Reference level		Reference level	
C2 *Non‐active Sleepers*	1.37 (0.6, 3.12)	0.456	1.57 (0.64, 3.83)	0.327
C3 *Screeners*	1.51 (0.64, 3.64)	0.350	1.7 (0.67, 4.33)	0.265

*Note:* In the adjusted models, adjusted for sex, location and parental education level.

Abbreviations: OR, odds ratio; CI, confidence interval.

## 4 Discussion

This is the first study to report clusters of Mongolian preschool‐aged children based on movement behaviours and explore their associations with eating behaviours and adiposity. We identified three distinct clusters (labelled as *All‐rounders*, *Non‐active Sleepers* and *Screeners*), which did not differ significantly in sociodemographic characteristics and adiposity levels. *Screeners*, however, demonstrated a greater tendency to consume unhealthy snacks and sugar‐sweetened beverages regularly compared with counterparts in the other two clusters.

### 4.1 Clustering of Movement Behaviours

Although we did not find clear‐cut categories of healthy and unhealthy clusters of movement behaviours, our findings did provide a more nuanced picture, revealing the presence of two mixed clusters and one relatively healthier cluster. This aligns with prior research showing that mixed lifestyle patterns were more prevalent (*n* = 40) than exclusively healthy (*n* = 26) or unhealthy (*n* = 31) patterns among school‐aged children (D'Souza et al. 2020). Another review on clustering patterns of PA and SB found that over 70% of the clusters identified among children and adolescents (0–19 years) contained at least one unhealthy behaviour (Mello et al. 2023). These findings suggest that children's movement behaviours tend to co‐occur in complex ways that go beyond ideal categorisations of healthy and unhealthy. This might be a result of the multitude of individual‐ and family‐level, and social and environmental factors influencing children's lifestyle behaviours (Rollo, Antsygina, and Tremblay 2020; Mello et al. 2023). Therefore, it is essential to examine movement behaviours in combination rather than in isolation to better inform the development of lifestyle intervention strategies.

Around half the children were classified into clusters with a mix of behaviours (*Non‐active Sleepers* and *Screeners*) and could be considered as high‐risk groups due to the potential deterioration of their movement behaviours as they age. For example, a longitudinal study of 123 Australian children aged 5–6 years found that children's behaviours became more unhealthy over time, with half the participants shifting clusters by ages 8–9, predominantly moving into the high SB/low MVPA cluster (Leech, McNaughton, and Timperio 2015). Similarly, a UK cohort study observed that 70% of participants transitioned to less healthy clusters characterized by higher SB between ages 6 and 9 (Jago et al. 2018). Collectively, these findings suggest that intervention efforts should begin in early childhood to establish and maintain healthy lifestyle behaviours and prevent the onset of obesity (Rollo, Antsygina, and Tremblay 2020; Kuzik et al. 2017).

### 4.2 Association of Cluster Membership With Eating Behaviours

The cluster of *Screeners* exhibited less healthy eating behaviours, possibly driven by the higher consumption of unhealthy snacks and sweetened drinks. Our findings align with a previous meta‐analysis, showing a strong and positive association between high levels of leisure‐time SB (> 3 h/day) and the consumption of fast food (OR = 1.97; 95% CI: 1.62–2.39) or soft drinks (OR = 1.31; 95% CI: 1.15–1.49) among Mongolian adolescents (Ashdown‐Franks et al. 2019). These results underscore the tendency of these two behaviours to co‐occur together.

The co‐occurrence of these behaviours in our study can be explained in two ways. First, it may be partly attributed to exposure to unhealthy food and beverage advertisements on TV. Reviews have shown that eating while watching TV is associated with increased consumption of sugar‐sweetened beverages and junk food, and fewer servings of fruits and vegetables among children (Avery, Anderson, and McCullough 2017; Scaglioni et al. 2018). Our results also showed that half of households turn on the TV or use another screen device during meals, further supporting this explanation. Second, the observed behaviour pattern can be explained by parental influence and family environment as young children's behaviour and lifestyle are mostly shaped by their parents or caregivers (Scaglioni et al. 2018). Previous research has shown that children's SB and TV‐watching are mainly shaped by parental practices such as their own TV‐watching, staying sedentary as well as monitoring child's screen time. Similarly, for eating behaviours, parents' knowledge of nutrition and their concern for health and disease prevention are directly linked to their children's dietary intake (Scaglioni et al. 2018).

Unfortunately, in Mongolia, there remains a gap in research investigating the relationship between SB, screen time and diet quality, especially in young children. However, existing evidence underscores a concerning trend: nearly half of Mongolian school children exhibit elevated consumption of high‐fat and high‐sugar food, a finding that aligns with the outcomes of our own study (Ashdown‐Franks et al. 2019; Nakahara et al. 2020). Compounding this issue is the high prevalence of prolonged recreational screen time and insufficient levels of PA among Mongolian children (Guthold et al. 2020; National Statistics Office of Mongolia, UNICEF 2019). Based on these findings, it is recommended that families implement strategies such as setting rules around screen time, ensuring the availability of healthy food options at home and practicing family mealtime without TV, which may help reduce the detrimental pattern of ‘sedentary‐snacking’ (Scaglioni et al. 2018).

### 4.3 Association of Cluster Membership With Adiposity

There was no difference in BMI z‐scores between clusters. However, clusters with a mix of behaviours were 1.7 times more likely to be overweight than the healthier cluster of *All‐rounders*, although this was not statistically significant. Similar findings were reported in a cross‐sectional study of 2090 3‐year‐old children in the Netherlands, which found that BMI z‐scores were not associated with any of the identified lifestyle patterns (Wang et al. 2020). However, two longitudinal studies have found that, while there was no cross‐sectional association between cluster membership and BMI z‐scores at a younger age of 5–6, follow‐up after 3 years showed that children who were more sedentary, had higher screen time, and engaging in more unhealthy snacking behaviours had significantly higher odds of being overweight or obese (Leech, McNaughton, and Timperio 2015; Gubbels et al. 2012).

The lack of associations in the present study can be partially explained by the limitations of the BMI‐z score for differentiating between fat mass and lean mass. Another possible explanation for our results may be related to the young age of our study population. The negative impact of unhealthy lifestyle behaviours on children's BMI and weight status may not be evident at this age, but they may accumulate over time and become apparent in later years. Furthermore, children may experience adiposity rebound around this age which can be another reason why we did not find any association with BMI z‐scores (Rolland‐Cachera et al. 1984).

Future research should aim to include a diverse range of adiposity indicators, such as waist circumference and body composition measures, in addition to BMI. This comprehensive assessment approach will provide more detailed and accurate results for adiposity. Additionally, longitudinal studies are needed to track the impact of movement behaviours on adiposity from early to middle childhood.

### 4.4 Strengths and Limitations

This is the first known study to examine clustering of movement behaviours in association with eating behaviours among Mongolian children. A strength of this study is the inclusion of all movement behaviours (PA, SB, sleep and screen time) in clustering, whereas most previous studies included only PA and SB (D'Souza et al. 2020). We also examined eating behaviours that provide important contextual information about diet of children such as having meals while TV is on, sitting together with parents or as a family during mealtimes.

Several limitations should be considered in interpreting the results of this study. The first limitation concerns a method of cluster analysis which derives cluster solutions from individual differences between participants. The choice of input variables plays a significant role in determining the final clustering outcome. Consequently, the results should be interpreted with caution and may not be generalizable to a broader population. Additionally, the study employed a convenience sampling approach and had a small sample size, limiting the generalisability of the findings and reducing the statistical power to detect meaningful associations.

## 5 Conclusion

This study revealed that Mongolian preschool children can be grouped into three distinct clusters based on their 24‐h movement behaviours. Half the children exhibited a mix of healthy and unhealthy behaviours, which may place them at higher risk of developing detrimental behaviours as they age. Our findings suggest that obesity prevention measures should begin in early childhood and target high‐risk clusters, considering the co‐occurrence of healthy and unhealthy behaviours.

Further research in Mongolia is necessary to provide evidence for obesity prevention policies and inform targeted interventions to promote healthy behaviours from an early age. Such studies should aim to include a diverse range of sociodemographic characteristics and health indicators and employ longitudinal designs to ensure a more comprehensive understanding of children's lifestyle behaviours in Mongolia.

## Author Contributions

**Ankhmaa Byambaa:** conceptualization, investigation, writing – original draft, methodology, visualization, writing – review and editing, formal analysis, project administration. **Rachel A. Jones:** writing – review and editing, supervision, methodology. **Kar Hau Chong:** writing – review and editing, formal analysis, data curation. **Oyundelger Dechinjamts:** writing – review and editing, project administration, investigation. **Bayasgalan Jambaldorj:** project administration, investigation, writing – review and editing. **Anthony D. Okely:** supervision, project administration, writing – review and editing, methodology, funding acquisition.

## Ethics Statement

The SUNRISE study was granted ethical approval from the Mongolian Ministry of Health's Medical Ethics Committee (ref.138, 26/12/2019; ref.23/057, 13/11/2023) and University of Wollongong's Human Ethics Research Committee (ref.2018/044; ref.2019/378).

## Conflicts of Interest

The authors declare no conflicts of interest.

## Supporting information


**Table S1** Questions used to assess eating behaviours.
**Table S2.** The sociodemographic distribution of participants by clusters.
**Figure S1.** Dendrogram of hierarchical clustering.
**Figure S2.** Cluster plot of k‐means clustering.
**Figure S3.** Final cluster centres by z‐scores.

## Data Availability

The data that support the findings of this study are available on request from the corresponding author. The data are not publicly available due to privacy or ethical restrictions.
