# Supplementary material for: Clustering of 24‐H Movement Behaviours and Its Associations With Eating Behaviours and Adiposity Among Mongolian Preschool Children: A Cross‐Sectional Study
Source: Child Care Health Dev. 2025 Apr 16;51(3):e70084. doi: 10.1111/cch.70084 (PMC12002562; doi:10.1111/cch.70084)
Supplement: Supplementary file 1 — Table S1 Questions used to assess eating behaviours. Table S2. The sociodemographic distribution of participants by clusters. Figure S1. Dendrogram of hierarchical clustering. Figure S2. Cluster plot of k‐means clustering. Figure S3. Final cluster centres by z‐scores. [file CCH-51-e70084-s001.docx]

**SUPPLEMENTARY MATERIALS**

**Table S1. Questions used to assess eating behaviours**

| **n** | **Questions** | **Response options** |
| --- | --- | --- |
| 1 | How often is the TV or an electronic screen device on during meal or snack time? | Never, Rarely, Once a week, Most days, Every day, Don’t know* |
| 2 | How often do you sit with your child during meals? |  |
| 3 | How often do all of the family sit together during a main meal? |  |
| 4 | How often does your child have snacks like chips, biscuits, cakes, candies, chocolate, pastries, and sweets between meals? |  |
| 5 | How often does your child drink sugary drinks (e.g. Cordials, flavoured milk, fruit juice, soda, soft drink)? |  |

* "Don't know" responses were treated as missing data and excluded from the analysis.

**Table S2. The socio-demographic distribution of participants by clusters**

|  | **Total** | **C1**  **All-rounders** | **C2**  **Non-active Sleepers** | **C3**  **Screeners** | **p-value** |
| --- | --- | --- | --- | --- | --- |
| **Sex (n=176)** | | | | | |
| Boys | 88 | 43 (48.9%) | 25 (28.4%) | 20 (22.7%) | 0.984 |
| Girls | 88 | 42 (47.7%) | 26 (29.5%) | 20 (22.7%) |  |
| **Sector (n=176)** | | | | | |
| Urban | 96 | 46 (47.9%) | 24 (25%) | 26 (27%) | 0.232 |
| Rural | 80 | 39 (48.8%) | 27 (33.7%) | 14 (17.5%) |  |
| **Location (n=176)** | | | | | |
| Ulaanbaatar | 107 | 56 (52.3%) | 24 (22.4%) | 27 (25.2) | 0.057 |
| Province | 69 | 29 (42%) | 27 (39.1%) | 13 (18.8%) |  |
| **Parental education level (n=173)** | | | | | |
| Full secondary and below | 45 | 22 (48.9%) | 12 (26.7%) | 11 (24.4%) | 0.922 |
| Tertiary and higher | 128 | 61 (47.7%) | 38 (29.7%) | 29 (22.7%) |  |

**Figure S1. Dendrogram of hierarchical clustering**

**
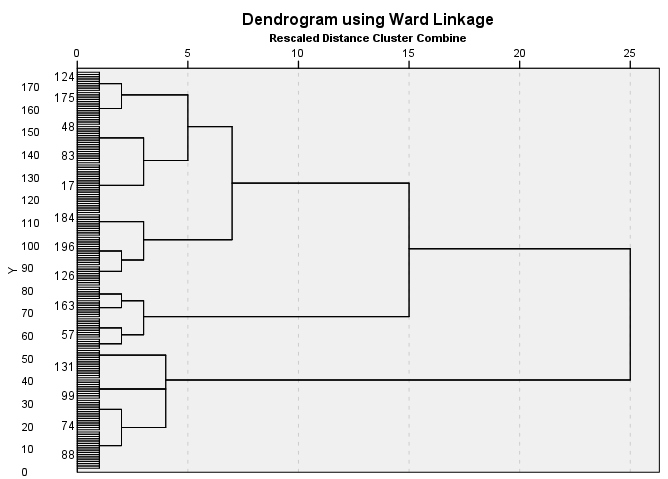
**

**Figure S2. Cluster plot of k-means clustering**

|  | |
| --- | --- |
|  | All-rounders |
|  | Non-active Sleepers |
|  | Screeners |


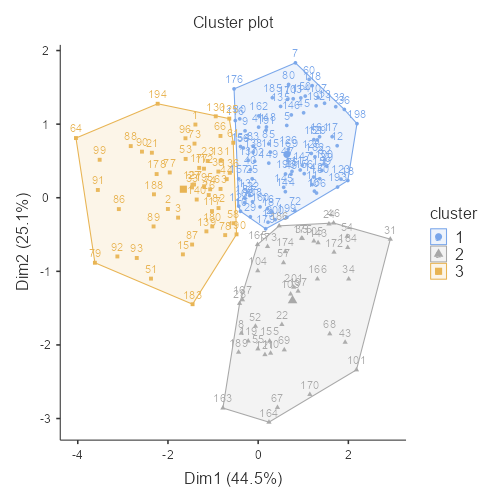


**Figure S3. Final cluster centres by z-scores**


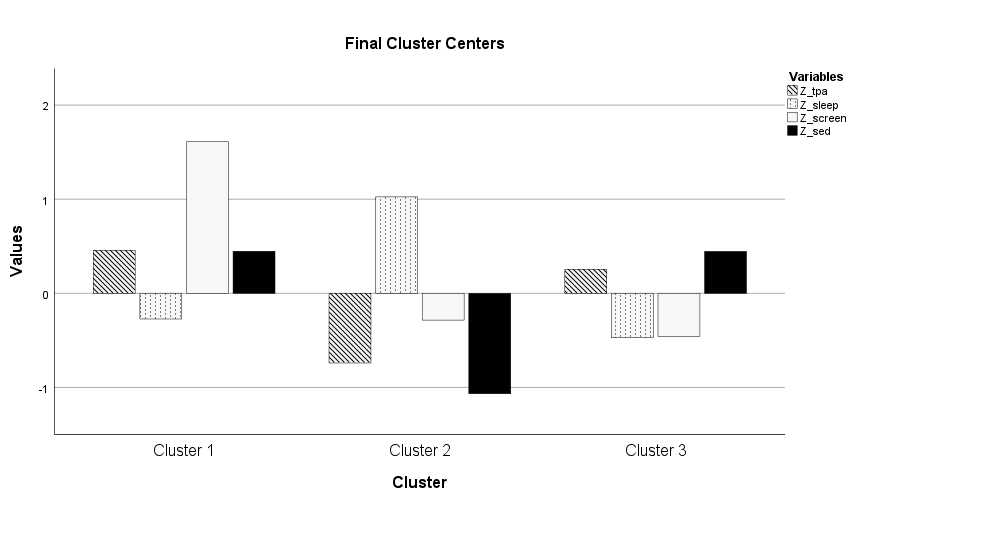


z-score

**PA**

**Sleep**

**Screen**

**SB**

**All-rounders**

**Non-active Sleepers**

**Screeners**

Abbreviation: PA – physical activity; SB – sedentary behaviour
